# ErbB4 promotes M2 activation of macrophages in idiopathic pulmonary fibrosis

**DOI:** 10.1515/biol-2022-0692

**Published:** 2023-10-03

**Authors:** Yu Jiang, Jialin Shi, Junhao Zhou, Chunxiao He, Ruinan Gu

**Affiliations:** School of Medicine, Shaoxing University, Shaoxing 312000, Zhejiang, China; Shaoxing Traditional Chinese Medicine Hospital, Shaoxing 312000, Zhejiang, China; Shaoxing People’s Hospital, Shaoxing 312000, Zhejiang, China

**Keywords:** ErbB4, IPF, M2 activation, alveolar macrophages, ERK

## Abstract

Idiopathic pulmonary fibrosis (IPF) is the most common and fatal diffuse fibrotic lung disease accompanied by macrophage M2 activation. ErbB4 is involved in and affects the process of inflammation. In this study, we determined that the mRNA level and protein expression of ErbB4 and M2 cytokine members were increased in the serum of IPF patients. In mouse alveolar macrophage MH-S cells, after knocking down ErbB4 by siRNA, the mRNA level and protein expression of M2 activator induced by interleukin (IL)-4 were decreased compared with the control group. Activating by ErbB4 agonist neuromodulatory protein (NRG)-1, IL-4-induced M2 program was promoted. Mechanistically, treated with NRG-1 in MH-S cells, the phosphorylation level of Akt did not change, while the phosphorylation level of ERK increased. Using SCH772984 to inhibit ERK pathway, the increasing IL-4-induced M2 activation by NRG-1 was inhibited, and the high level of M2 activator protein expression and mRNA expression was restored. Collectively, our data support that ErbB4 and M2 programs are implicated in IPF, and ErbB4 participates in the regulation of M2 activation induced by IL-4 through the ERK pathway.

## 1 Introduction

Idiopathic pulmonary fibrosis (IPF) is the most common and fatal diffuse fibrotic lung disease, which can lead to progressive loss of lung function [1]. In recent years, the incidence rate of IPF has increased significantly, the mortality rate has increased, the average survival time after diagnosis is only 2.8 years, and its mortality rate exceeds many cancers, which has become a serious social health problem [2]. As a disease with unknown etiology and poor prognosis, IPF has few specific therapeutic drugs at present. Recently, there are some new methods for the treatment of pulmonary diseases oxygenation with nanobubbles and gene editing-based methods applicable for early diagnosis/therapy [3,4,5]. In a word, it is essential to find a new therapeutic target of IPF. All stages of pulmonary fibrosis are accompanied by innate and adaptive immune responses [6]. The transformation of lung tissue from acute inflammatory injury to chronic pulmonary fibrosis is accompanied by the transformation of macrophages from M1 type to M2 type, and finally, M2 type with anti-inflammatory and pro-fibrosis effects is the main type [7]. M2 macrophages are involved in the occurrence and development of IPF and play a key role in the process of fibrosis [8].

ErbB4 is a member of the ErbB family of tyrosine kinase receptors. It can bind and be activated by neuromodulatory protein (NRG) and other factors and induce a series of cellular responses such as cell division and cell differentiation [9]. The main signaling pathways of ErbB4 activation include immune regulation, RAS-MAPK-ERK pathway, and PI3K-Akt pathway [10]. ErbB4 is involved in the occurrence and development of many diseases and affects the regulation of the inflammatory process [11]. Endogenous NRG4-ErbB4 signaling limits the macrophage production of proinflammatory cytokines *in vitro* and limits colitis severity *in vivo* [12]. Severe inflammation and M1 macrophage polarization were detected in the inguinal and epididymal white adipose tissues in ErbB4 deletion mice [13]. Activation of ErbB4 can induce macrophage apoptosis in a mouse model of inflammatory bowel disease, thereby inhibiting intestinal inflammation [14].

Macrophage M2 activation is involved in the occurrence and development of IPF and plays a key role in the process of fibrosis, while ErbB4 is involved in and affects the process of inflammation. However, whether ErbB4 regulates M2 activation of macrophages or participates in the occurrence and development of IPF related to M2 activation of macrophages was not investigated. In this study, we characterized the expression of ErbB4 in the serum of patients with IPF and the effect of ErbB4 on M2 activation of macrophages.

## 2 Materials and methods

### 2.1 Human samples

Sixty patients with pulmonary fibrosis treated in the hospital from January 2020 to January 2022 were selected: 36 males and 24 females aged from 51 to 73 years. IPF was diagnosed according to the American thoracic society/European respiratory society consensus diagnostic criteria. The liver and kidney functions of the patients were normal. All patients signed an informed consent. This study was discussed and approved by the hospital ethics committee and passed the ethical approval. Collection of human serum samples: 5 mL of serum samples from normal adults and patients with confirmed pulmonary fibrosis was collected, centrifuged, and stored in a low-temperature refrigerator at −80°C. All samples were collected and kept at hospital.


**Informed consent:** Informed consent has been obtained from all individuals included in this study.
**Ethical approval:** The research related to human use has been complied with all the relevant national regulations, institutional policies, and in accordance with the tenets of the Helsinki Declaration, and has been approved by hospital ethics committee.

### 2.2 Cell culture

Mouse alveolar macrophage MH-S cell is a kind of suspension cell cultured in a 37°C, 5% CO_2_ cell incubator with 1640 medium (90% Roswell park memorial institute-1640 + 10% fetal bovine serum). MH-S cells were induced to differentiate into M2 by treating with 20 ng/mL interleukin (IL)-4 (PeproTech) for 48 h. ErbB4 agonist NRG-1 was purchased from Solarbio (P00442). MH-S cells were treated with 10 nm NRG-1 for 12 h. ERK inhibitor SCH772984 was purchased from Abmole Bioscience (m2084). MH-S cells were treated with 1 nm SCH772984 for 12 h.

### 2.3 Quantitative real-time polymerase chain reaction (PCR)

RNA was extracted from 250 μL serum and MH-S cells isolated using TRIzol Reagent (Invitrogen) according to the manufacturer’s instructions. Quantitative reverse transcription-polymerase chain reaction analysis was performed using SYBR Green Master (Roche). The relative expression for each target gene was normalized by β-actin. The following primers were used for each target gene: β-actin, 5′-AGCCATGTACGTAGCCATCC-3′, 5′-TCTCAGCGTGGTGGTGAAG-3′; ErbB4, 5′-GGAATATTTGGTCCCCCAGGCTTTC-3′,5′-GAGGAGGGCTGTGTCCAATTTCAC-3′; human Arg-1, 5′-CAGAAGAA TGGAAGAGTCAG-3′, 5′-CAGATATGCAGGGAGTCACC-3′; human YM-1, 5ʹ-TGAGGAAGAATCTGTGGAGAA-3ʹ, 5ʹ-TGAGACAGTTCAGGGATCTTG-3ʹ; human transforming growth factor (TGF)-β, 5ʹ-TGCCCTCCTACGGACTTGA-3ʹ,5ʹ-GCTGAGAACCCTGCTATGCT-3ʹ; mouse Arg1, 5ʹ-GCCAGGGACTGACTACCTTAA-3ʹ, 5ʹ-AGTTCTGTCTGCTTTGCTGTG-3ʹ; mouse YM-1, 5ʹ-TGGAGGATGGAAGTTTGGAC-3ʹ, 5ʹ-AATGATTCCTGCTCCTGTGG-3ʹ; mouse TGF-β, 5ʹ-GCGGCAACCAAATCTATGA-3ʹ, 5ʹ-GTGGGCACTGAGGCGAAAA-3ʹ.

### 2.4 Enzyme-linked immunosorbent assay (ELISA)

Human-ErbB4, human-IL-10, mouse-IL-10, human-TGF-β, and mouse-TGF-β in conditioned cell culture media were measured using ELISA kits (FN-EH8285; FN-EH0173; FN-EM0100; FN-EH0287; FN-EM0176) from Wuhan Fine Biotech following the manufacturer’s instruction.

### 2.5 SiRNA transfection

The MH-S cell line was cultured in cell bottle (25 cm^2^) for 24 h and transfected with siRNA by Lipofectamine® Rnaimax (ErbB4 siRNA: 5′-CCCAAACAA GAATACCTG aat-3′; control: 5′-TTCTCCGAACGTGTCACG T-3′). After 48 h, the transfection efficiency was detected by real time quantitative PCR and western blot.

### 2.6 Western blot

Cultured cells were homogenized in lysis buffer, protein samples were loaded onto sodium dodecyl sulfate-polyacrylamide gels, and run in running buffer. Proteins were transferred onto methanol-activated polyvinylidene difluoride membranes in transfer buffer. After incubating with primary antibodies and secondary antibody, the membranes were developed using the Bio-Rad ChemiDoc imaging system. A-tubulin, ErbB4, p-ErbB4, Akt, p-Akt, ERK, and p-ERK antibodies were purchased from cell signaling.

### 2.7 Statistical analysis

Comparisons between groups were undertaken using the GraphPad Prism6 software. Two experimental groups were compared using *t*-test for paired data. For comparison between more than two groups, a one-way analysis of variance was used. The data were presented as the mean ± standard error of mean. In all cases, *p* < 0.05 was considered significant. *p < 0.05; **p < 0.01.

## 3 Results

### 3.1 ErbB4 expression is increased in patients with IPF

**Figure 1:**
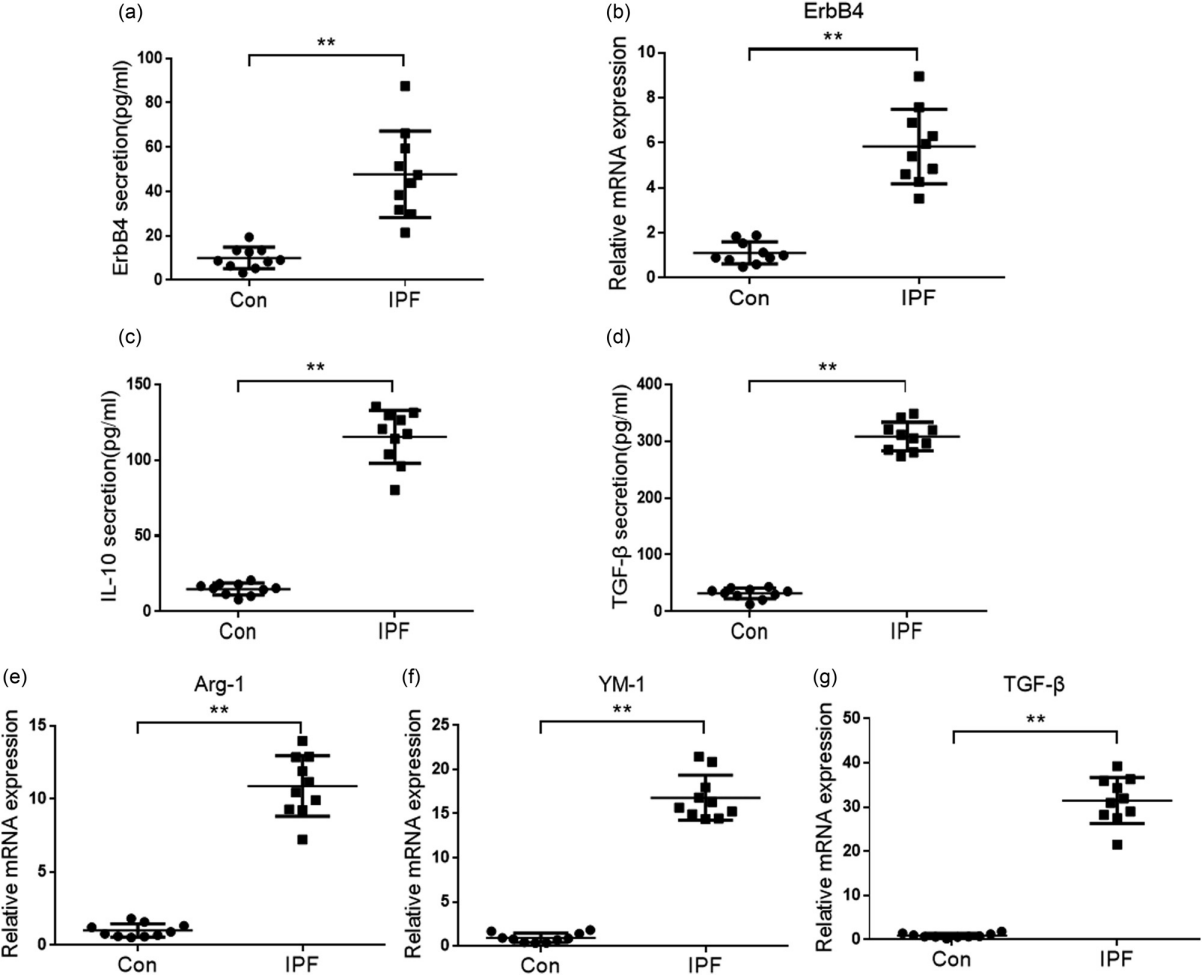
Expression of ErbB4 and M2 activator in the serum of patients with IPF. Detecting the protein expression of ErbB4 (a), IL-10 (c), and TGF-β (d) in the serum of IPF patients by ELISA kit. Detecting the mRNA level of ErbB4 (b), Arg-1 (e),YM-1 (f), and TGF-β (g) in the serum of IPF patients by qPCR. *n* = 10, **, *p* < 0.01.

We first detected the expression level of ErbB4 in the serum of patients with IPF. ELISA showed that the content of ErbB4 protein in serum of the normal group was lower, while the content of ErbB4 protein in serum of patients with IPF was significantly higher (Figure 1a). The mRNA level of ErbB4 in serum was detected by quantitative real-time PCR (qPCR). It was also found that the mRNA level of ErbB4 in serum of IPF patients was higher than that of the normal group (Figure 1b). M2 macrophages are mainly involved in the occurrence and development of IPF; then, we detected the expression of M2-activating factors IL-10, Arg-1, YM-1, and TGF-β in serum. Compared with the normal group, the protein levels of IL-10 and TGF-β in the serum of IPF patients were increased (Figure 1c and d), so as the mRNA level of Arg-1, YM-1, and TGF-β (Figure 1e–g). The results showed that the occurrence and development of IPF was accompanied by M2 activation of macrophages and increased expression of ErbB4.

### 3.2 SiErbB4 inhibits the M2 activation of MH-S cells induced By IL-4

**Figure 2:**
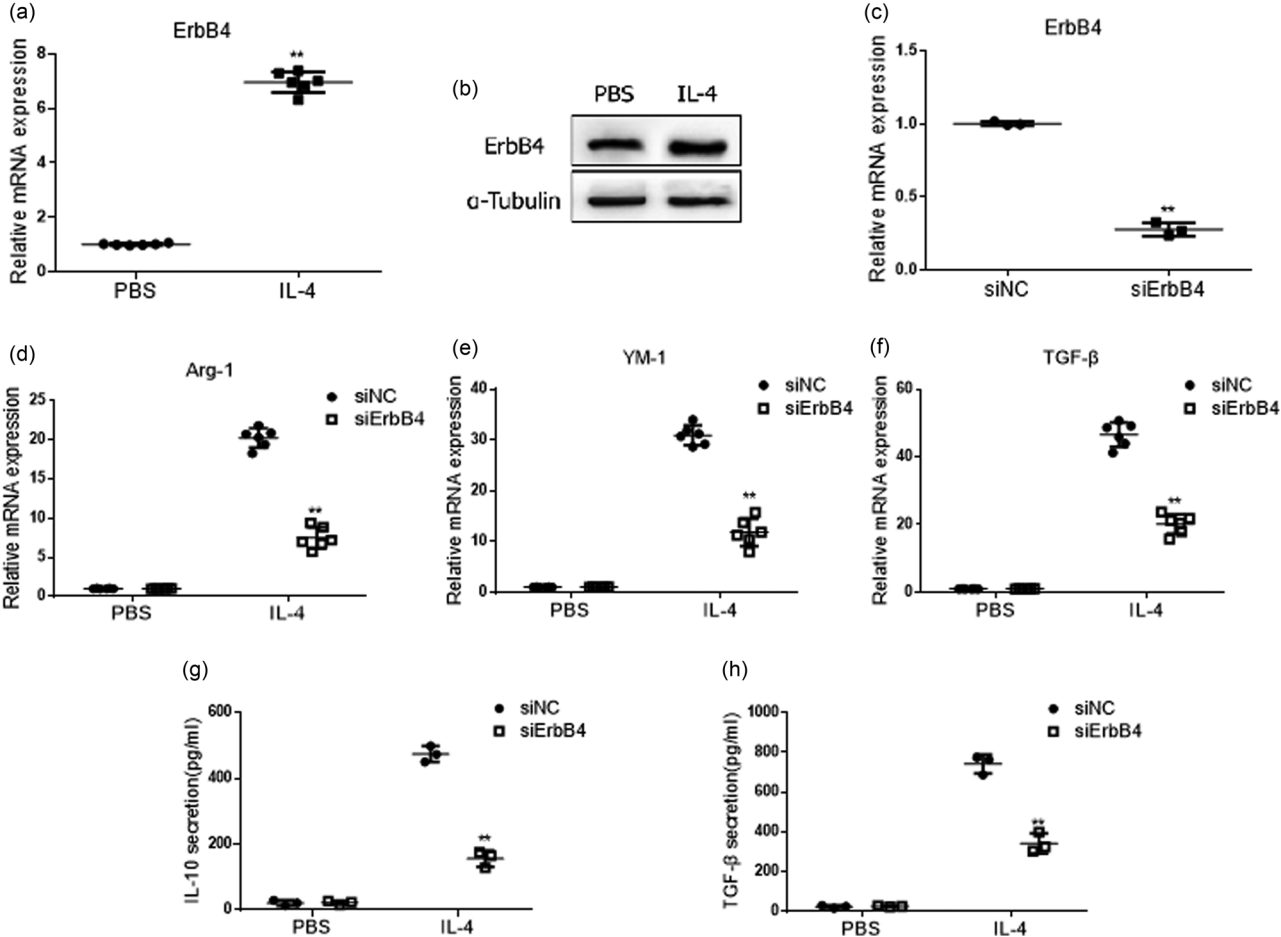
M2 activation of MH-S cells after knocking down ErbB4. Treated with IL-4 for 48 h, the mRNA level of ErbB4 was detected by qPCR (a), and the protein expression of ErbB4 was detected by western blot in MH-S cells (b). ErbB4 siRNA was transfected into MH-S cells, and the level of ErbB4 mRNA was detected by qPCR (c). Treated with IL-4 for 48 h,the mRNA level of Arg-1,YM-1, and TGF-β was detected by qPCR (d–f), and the protein expression of IL-10,TGF-β was detected by ELISA kit in MH-S cells (g and h). *n* = 3, **, *p* < 0.01.

To explore the effect of ErbB4 on M2 activation of macrophages, we used mouse alveolar macrophage MH-S cells for study. After treating with IL-4 for 48 h in MH-S cells, qPCR and western blot showed that the mRNA level and protein content of ErbB4 increased (Figure 2a and b). siRNA was transfected into MH-S cells to knock down ErbB4 expression (Figure 2c). After treating with IL-4 for 48 h, the expressions of M2-activating factors IL-10, Arg-1, YM-1, and TGF-β were detected. The results showed that the decrease in ErbB4 expression did not cause M2 activation of MH-S cells; however, after IL-4 stimulation, the mRNA level of Arg-1, YM-1, and TGF-β (Figure 2d–f) and the protein expression of IL-10 and TGF-β (Figure 2g and h) in siErbB4 MH-S cells were significantly decreased compared with the control group. It suggested that knocking down the expression of ErbB4 in MH-S cells inhibits the M2 activation of alveolar macrophages induced by IL-4.

### 3.3 Activation of ErbB4 increases IL-4-induced M2 activation

**Figure 3:**
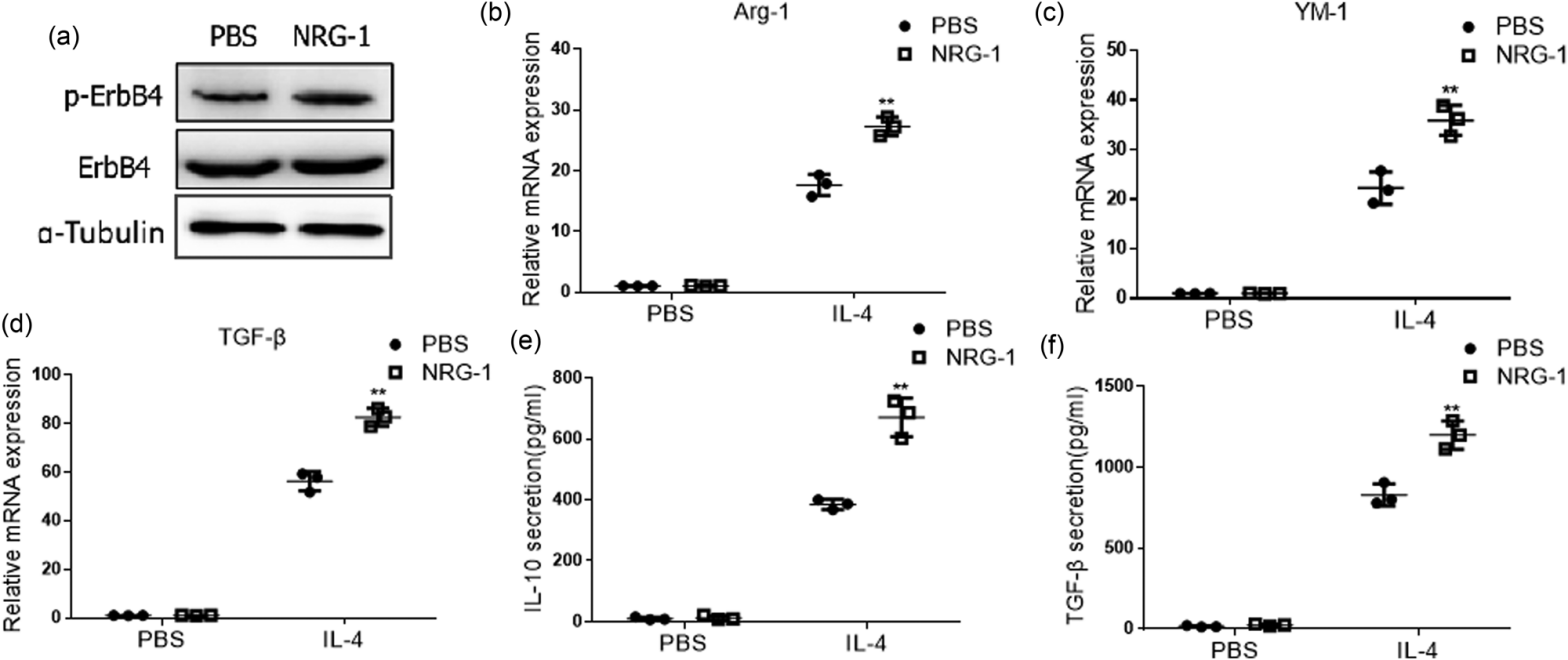
M2 activation of MH-S cells treated with NRG-1. Treated with NRG-1 for 12 h, the protein expression of ErbB4 was detected by western blot in MH-S cells (a). Treated with NRG-1 for 12 h and another 48 h with IL-4, the mRNA level of Arg-1,YM-1, and TGF-β was detected by qPCR (b–d), and the protein expression of IL-10 and TGF-β was detected by ELISA kit in MH-S cells (e–f). *n* = 3, **, *p* < 0.01.

To further investigate the effect of ErbB4 on M2 activation, we used ErbB4 agonist NRG-1 to activate ErbB4. After treating with NRG-1 for 12 h and IL-4 for 48 h, the expressions of M2-activating factors IL-10, Arg-1, YM-1, and TGF-β in MH-S cells were detected. Western blot showed that NRG-1 could increase the phosphorylation level of ErbB4 (Figure 3a), but did not cause the M2 activation of MH-S cells. However, after stimulating by IL-4, compared with the control group, the mRNA level of Arg-1, YM-1, and TGF-β (Figure 3b–d) and the protein expression of IL-10 and TGF-β (Figure 3e and f) were markedly increased in MH-S cells treated with NRG-1. The results suggest that the activation of ErbB4 in MH-S cells can increase IL-4-induced M2 activation. The aforementioned results suggest that ErbB4 participates in and affects M2 activation of alveolar macrophages.

### 3.4 ErbB4 regulates macrophage M2 activation through the ERK pathway

**Figure 4:**
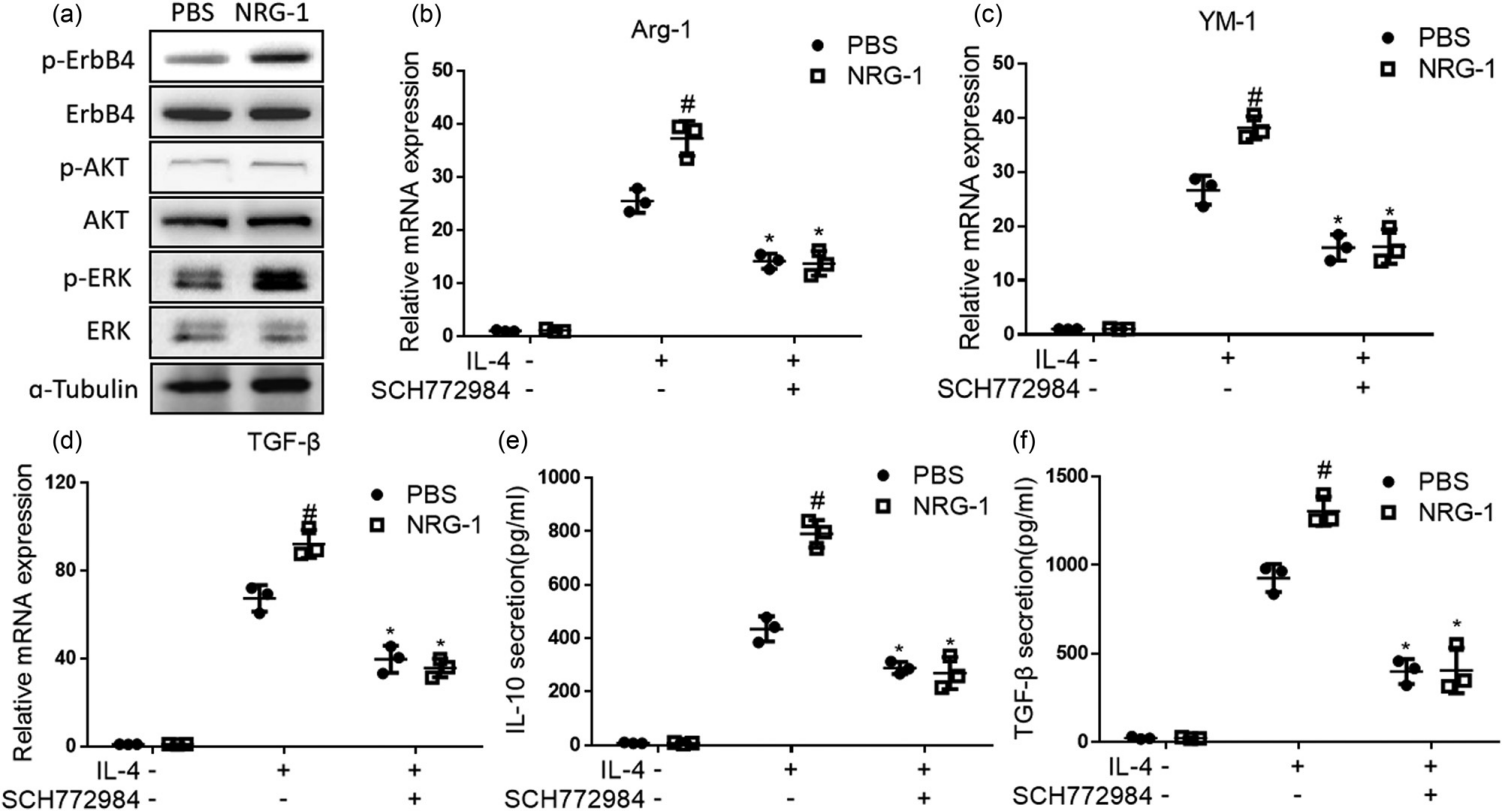
Treated with NRG-1 for 12 h, the protein expression of p-AKT and p-ERK was detected by western blot in MH-S cells (a). Treated with NRG-1 and SCH772984 for 12 h and another 48 h with IL-4, the mRNA level of Arg-1,YM-1, and TGF-β was detected by qPCR (b–d), and the protein expression of IL-10 and TGF-β was detected by ELISA kit in MH-S cells (e and f). *n* = 3, **, *p* < 0.01.

Akt and ERK are the main downstream targets of ErbB family kinases [15]; therefore, we first studied the correlation between ErbB4 function and their activities. We used ErbB4 agonist NRG-1 to explore the changes of Akt and ERK phosphorylation after ErbB4 activation in MH-S cells. MH-S cells were treated with NRG-1 for 12 h; the protein levels of p-Akt and p-ERK were detected by western blot. It was found that the phosphorylation level of Akt did not change, but the phosphorylation level of ERK increased (Figure 4a). It shows that ErbB4 activation does not affect the Akt pathway but through the ERK pathway in MH-S cells. Next, we used ERK inhibitor SCH772984 to further explore the role of p38 MAPK pathway in the effect of ErbB4 on M2 activation of macrophages. MH-S cells were treated with NRG-1 and SCH772984 for 12 h and then treated with IL-4 for 48 h; the changes of expression of M2-activating factors IL-10, Arg-1, YM-1, and TGF-β were measured. The results showed that after IL-4 stimulation, SCH772984 blocked the increased mRNA levels of Arg-1, YM-1, and TGF-β (Figure 4b–d) and the protein levels of IL-10 and TGF-β (Figure 4e and f) increased by NRG-1. It suggests that ErbB4 was involved in the regulation of IL-4-induced M2 activation through the ERK pathway in MH-S cells.

## 4 Discussion

Pulmonary fibrosis is an irreversible pathological process in which normal lung tissue is replaced by fibroblasts or collagen. It is characterized by fibroblast proliferation, accumulation of a large number of extracellular matrix, accompanied by inflammatory injury, and destruction of tissue structure. It eventually leads to lung scars, structural distortion, and irreversible loss of function. It is a chronic, progressive, and fibrotic interstitial lung disease [1]. As a common and fatal disease, the pathogenesis and effective therapeutic drugs of IPF need to be overcome urgently. There are some relations between IPF and corona virus disease 2019 (COVID-19). The major risk factors for severe COVID-19 such as increasing age, male sex, and comorbidities (such as hypertension and diabetes) are shared with IPF [16]. A positive genetic correlation of IPF with COVID-19 severity was detected, and genetically increased risk of IPF has a causal effect on COVID-19 severity [17]. As the further research of IPF and COVID-19, there are some new methods with nanobubbles for the treatment of pulmonary diseases oxygenation in COVID-19 [3]. On the other hand, there are some gene editing-based methods applicable for early diagnosis/therapy of diverse cancers, before the appearance of the clinical complications [4,5]. Using the clinical data and clinical samples of patients, this study explored the relationship between ErbB4, IPF, and macrophage activation in the serum of patients with IPF and studied the effect of ErbB4 on macrophage M2 activation in mouse alveolar macrophage MH-S cells, to provide a reference for the early diagnosis and later disease development control of patients with IPF. It also provides a basis for the follow-up study on how ErbB4 participates in the occurrence and development of IPF by affecting the M2 activation of macrophages.

Alveolar macrophage (AM) is a specific phagocyte located on the surface of the lower respiratory tract. As the first line of immune defense of the lung, it can respond quickly after lung tissue injury or pathogen invasion. Although the exact etiology of IPF is unclear and diverse, all stages of fibrosis are accompanied by innate and adaptive immune responses [6]. The abnormal secretion of cytokines caused by persistent inflammatory reaction is an important pathological basis of pulmonary fibrosis [2]. Repeated lung tissue or alveolar epithelial cell injury leads to chronic inflammation, which eventually leads to excessive wound repair and tissue remodeling, resulting in fibrosis. M2 macrophages are involved in the occurrence and development of IPF and play a key role in the process of fibrosis. IL-13 is the main cytokine in fibrosis. Studies have found that M2 macrophages widely exist in fibrotic lungs. The serum concentration of various growth factors secreted by M2 macrophages, such as vascular endothelial growth factor, is closely related to the clinical and biological parameters of disease severity. Clearing M2 macrophages in the stage of pulmonary fibrosis can reduce the deposition of pulmonary extracellular matrix [18]. Our study found that the mRNA and protein levels of macrophage type M2-activating factors IL-10, Arg-1, YM-1, and TGF-β increased in the serum of patients with IPF, indicating the activation of M2 macrophages in patients with IPF, which is consistent with previous studies.

Phenotypic changes of pulmonary macrophage subsets are one of the characteristics of the occurrence and development of pulmonary fibrosis, accompanied by each process of pulmonary fibrosis. It is suggested that we can not only be used as a reference for diagnosis, but also as a new therapeutic target and means to prevent and treat pulmonary fibrosis. At present, there are many potential drugs for the treatment of pulmonary fibrosis in phase II/III clinical trials, such as the development of anti-pulmonary fibrosis drugs acting on targets such as inflammation, oxidative stress, alveolar epithelial cells, and myofibroblasts. Anti-inflammatory drugs are also the focus of research and development [19]. At present, studies have shown that glucocorticoids or combined immunosuppressants have clinical effects in the treatment of interstitial lung diseases dominated by inflammatory changes.

Tyrosine kinase receptor ErbB family controls cell proliferation and cell differentiation in many cells and tissues from epithelium, mesenchymal, and neurons. It plays an important role in the development and maturation of various organ systems such as lung, heart, and central nervous system [20]. ErbB4 is overexpressed in a variety of epithelial tissue-derived malignancies, such as lung cancer, ovarian cancer, prostate cancer, bladder cancer, and cervical cancer, which suggests that the change of ErbB4 expression is closely related to the occurrence and evolution of tumors. Adding a monoclonal antibody of ErbB4 to non-small-cell lung cancer cells expressing ErbB4 can inhibit cell growth and promote cell apoptosis [21], deletion of ErbB4 can result in proliferation of human breast cancer cells [22], and these all indicate that ErbB4 has a tumor suppressive effect. ErbB4 can promote the synthesis of alveolar surfactant and promote the maturation of lung [12]. In normal lung tissue, ErbB4 is expressed in bronchial mucosal epithelial cells and alveolar epithelial cells. In lung cancer, ErbB4 is overexpressed, and its localization is transferred from the cell membrane and cytoplasm to the nucleus and cytoplasm. The overexpression of ErbB4 may be related to lung cancer lymph node metastasis, tumor node metastasis staging, and postoperative survival rate [23]. ErbB3, another member of EGFR family, is also unique in that its tyrosine kinase domain is functionally defective. Studies of EebB3 expression in primary cancers have implicated with varying degrees of many cancers. Recent results link high ErbB3 activity with lung and breast cancers [24,25]. Somatic alterations with ErbB3 gene were identified in the tissue of lung cancer associated with IPF [26].

There are few studies on the effect of ErbB4 on pulmonary inflammation and fibrosis. Studies have shown that lapatinib, an ErbB inhibitor, can prevent the downstream of ErbBs (p38 MAPK, MEK/ERK, and Akt/mTOR), the production of proinflammatory cytokines, and the activation of epithelial barrier injury-related pathways in severe acute respiratory syndrome coronavirus 2-induced non-infectious acute lung injury and fibrosis [27]. A study of only five patients with IPF showed the top most upregulated genes with DCLK1, PDK4, and ErbB4 [28], which were consistent with the result in Figure 1 that the upregulated level of ErbB4 mRNA showed significant differences. There is a new class of pyrimidine derivatives that exhibited high inhibitory potency toward ErbB4 identified as inhibitors for the treatment of IPF [29]. The antifibrotic effect of NRG-1 in the lung is linked to anti-inflammatory activity NRG-1/ErbB4 signaling in macrophages [30].

Our study found that the mRNA and protein levels of ErbB4 in the serum of patients with IPF increased, suggesting that ErbB4 is involved in the occurrence and development of IPF, but the specific role of ErbB4 in the process of IPF needs to be further explored. In addition, we also found that the mRNA and protein levels of ErbB4 increased in the M2 activation of AMs induced by IL-4. After knocking down ErbB4, the M2 activation of macrophages was inhibited, and the activation of p-ErbB4 by agonists could increase the M2 activation induced by IL-4, suggesting that ErbB4 is related to the M2 activation of macrophages. We speculate that ErbB4 may be involved in the occurrence and development of IPF by regulating the M2 activation of macrophages. Transforming growth factor-β (TGF-β) is closely related to the occurrence and development of pulmonary fibrosis [31]. The level of TGF-β in the lungs of IPF patients increased. Our study also found that the mRNA and protein levels of TGF-β in the serum of IPF patients increased. Activating ErbB4 phosphorylation can increase the release of TGF-β induced by IL-4, which may be a mechanism for ErbB4 to increase the M2 activation of macrophages and further aggravate fibrosis.

In tumor cells, the main intracellular pathways associated with ErbB4 are Ras-MAPK-ERK and PI3K-Akt pathways [22]. In human catecholaminergic cells, ErbB4 can regulate the homeostasis of extracellular dopamine and norepinephrine, and ErbB4 can regulate extracellular dopamine through the p38 MAPK signaling pathway [32]. In neural progenitor cells, NRG1 can activate Akt phosphorylation and increase neuronal migration activity [12]. In inflammatory bowel disease, ErbB4 is involved in the regulation of inflammatory M1/M2 transformation through the interaction with STAT1 and STAT5 [11]. We used NRG-1 to activate ErbB4 in mouse alveolar macrophage MH-S cells and detected the downstream ERK and Akt pathways. It was found that activating ErbB4 can increase ERK phosphorylation activation without affecting Akt phosphorylation, indicating that Akt pathway in macrophages is not the main downstream pathway of ErbB4. After inhibiting ERK activation with SCH772984, the increase of macrophage M2 activation caused by NRG-1 was canceled and returned to a similar level as that without NRG-1, suggesting that ErbB4 may be involved in mediating il-4-induced macrophage M2 activation through the ERK pathway.

In conclusion, the expression of ErbB4 is increased in the serum of patients with IPF, and it participates in and affects the M2 activation of AMs through the ERK pathway. How ErbB4 participates in regulating the occurrence and development of IPF by affecting the M2 activation of macrophages is the direction of our future research. This proposition will lay a foundation for the clinical diagnosis and treatment of pulmonary inflammation and M2 activation of pulmonary macrophages and provide a theoretical support for ErbB4 as a new target for the diagnosis and treatment of inflammation and IPF.
